# Views Ancient and Modern on Placenta Praevia
*An abridged version of a paper given before the Bath, Bristol and Somerset Branch, B.M.A., on 6th February, 1952, at Royal United Hospital, Bath.


**Published:** 1952-04

**Authors:** John Stallworthy

**Affiliations:** Director, Department of Obstetrics and Gynaecology, Oxford United Hospitals


### VIEWS ANCIENT AND MODERN ON PLACENTA PRAEVIA*

BY

JOHN STALLWORTHY, F.R.C.S., F.R.C.O.G.

*Director, Department of Obstetrics and Gynaecology, Oxford United Hospitals*

Something of the part played by the placenta in the causation of antepartum haemorrhage was known as far back as the time of Hippocrates. The early fathers of medicine believed that if the placenta was found over the internal os, it had fallen there from its normal position.

In the seventeenth century Ambrose Pare taught and practised version and breech delivery as the treatment for antepartum haemorrhage, although at that time the cause of the bleeding was not fully understood. Pare's own daughter developed bleeding early in labour, but was so successfully treated in accordance with her father's teaching by one of his pupils, Guillemeaux, that both mother and baby survived.

It was not until 1775 that the two chief causes of antepartum haemorrhage were recognized. They were described by Rigby who analysed twenty-eight cases he had personally treated. In nine of these he had found the placenta attached to what he described as the zone of dilatation of the uterus. This is what we would now describe as the lower segment. He pointed out that if a placenta was attached to this zone, then it was inevitable that bleeding would occur during the dilatation of this zone in labour. He therefore called bleeding due to this cause " unavoidable haemorrhage ". This was the first accurate description of placenta praevia. It is interesting that of the nine cases he treated, five mothers died. In the remaining nineteen cases he found that the placenta was normally situated, and concluded that if bleeding occurred under these circumstances it was due to some accident or the result of violent emotional disturbances such as fear, anger, or passion. Rigby treated the cases of placenta praevia by version and breech delivery, but when the placenta was normally situated he advocated rupture of the membranes. This was so successful that in his nineteen cases he had no maternal deaths.

This tradition was carried on and reinforced by Ingleby of Birmingham early in the nineteenth century. He stressed mainly the importance of an accurate diagnosis at the earliest possible moment, and for this advocated careful exploration of the uterus after inserting the whole hand into the vagina and two fingers to their maximum length into the uterine cavity.

From 1800 until recent times, progress was slow, but two very important aids to treatment were increasingly practised. In 1820, the *Lancet* for the first time recorded the use of blood transfusion in the treatment of placenta praevia. Twelve ounces of blood from a coal heaver husband were successfully given to

* An abridged version of a paper given before the Bath, Bristol and Somerset Branch. B.M.A., on 6th February, 1952, at Royal United Hospital, Bath.

his wife. In a lecture at Guys Hospital at that time, a Dr. Blundell discussed the relative merits of animal and human blood donors, and pointed out the practical difficulties of obtaining the co-operation of suitable animals for transfusion in emergencies. The second development was the increasing use being made of Caesarean Section, first of all the classical operation and later the lower segment technique.

Progress continued on these lines until in 1945 Macafee of Belfast and Johnson of America challenged the dictum that an immediate vaginal examination should be made in antepartum haemorrhage. They pointed out that with proper hospital supervision, patients in whom placenta praevia was suspected could be nursed until the child was viable. In this way premature labour induced by vaginal examination was avoided with a consequent great reduction in foetal mortality. Meanwhile, Marshall at Liverpool was practising Caesarean Section in most cases of suspected placenta praevia.

Various classifications of the types of placenta praevia had been made by British and American authorities, but all were based on vaginal examination and the estimation of the relationship of the placenta to the internal os. The disadvantage of all these was that in the attempt to decide on the correct classification of a placenta praevia, haemorrhage was often induced with danger to both the mother and babe.

In Oxford the problems of antepartum haemorrhage and of placenta praevia in particular have been studied with great care after a difficult case which ended with a maternal death in 1940. In the later years of the investigation the clinical team was greatly assisted by Dr. Reid of the Department of Radiology who was developing his own technique for soft tissue radiography. Possibly even more important than this was the use he made of gravity to determine by inference whether the placenta was interfering with the engagement of the presenting part at the brim of the pelvis. Particular care was paid to the distance on the X-ray film between the head and the promontory of the sacrum behind and the symphisis pubis in front. This relationship was studied by two lateral films, one taken with the patient in the erect position, and one when she was tipped to an angle of sixty degrees. This was known as the semi-erect film. In most cases, with careful clinical assessment of the case, the radiological findings merely confirmed what the clinician has suspected from his abdominal examination. It had been learned that the most dangerous type of placenta praevia was the one which was attached posteriorly to the lower segment, and by overlying the promontory of the sacrum reduced the effective conjugate at the brim. In this way the presenting part was displaced from the brim, and malpresentations were common. In these cases it was also frequently found that the umbilical cord was attached to the lower pole of the placenta and when this was so, even if the presenting part did enter the brim, there was danger of pressure obstructing the foetal circulation and causing intra-uterine death.

The result of this study led to the belief that there were two safe ways of treating placenta praevia. The first was by rupturing the membranes and the second was by Caesarean Section. The choice of which method to use was made by assessing the relationship of the presenting part to the brim of the pelvis. If it could be made to enter, the membranes were ruptured, and if it could not, section was performed. This was done without any reference to the position of the placenta in terms of the internal os, and was a departure from orthodox views on the subject. When possible treatment was postponed until after the 36th week.

A most interesting and helpful colour film of the X-ray technique and lower segment Caesarean Section was then shown. Mr. Stallworthy then summarized the principles of treatment:

Firstly, that the possibility of placenta praevia should be diagnosed in the ante-natal clinic before haemorrhage began by the position of the head. A single X-ray in the semi-erect position was normally sufficient to indicate the possibility or otherwise of placenta praevia by the Reid technique. If haemorrhage began before thirty-six weeks the patient was admitted to hospital with all emergency equipment immediately available and could very frequently be carried along almost to full term with excellent prospects of obtaining a live child. If haemorrhage began after thirty-six weeks the child was viable and the course of action outlined above could be followed.

The great danger of moving such cases to a hospital was recognized, and in this connection there was no doubt that the " Flying Squad " which Mr Stallworthy, at the suggestion of Professor Chassar Moir, formed in 1939 had more than repaid the trouble and expense involved. It had now been called more than 400 times, and with only one death. On three occasions emergency Caesarean operations had to be undertaken in the nearest small hospital.

On an analysis of the latest figures, Mr. Stallworthy was able to show that in over 250 cases dealt with since the maternal death in 1940, no mother had died, and in the last 100 cases the uncorrected infant mortality including stillbirths and neonatal deaths had been ten.

### DISCUSSION

**Dr. H. M. Golding** (Chairman) stated how greatly the meeting had appreciated Mr. Stallworthy's address and invited comments and questions.

**Professor Lennon** paid a sincere tribute to the work and persistence of Mr. Stallworthy and Dr. Reid and stressed the vital importance of team work in dealing with these cases. He drew attention to the danger of cases in which the cord was attached eccentrically to the placenta so that an endeavour to pass the head into the pelvis could stop a foetal heart in a very short time. He also announced that a Flying Squad had begun operating in Bristol on 1st January and had already been in action.

**Dr. Sutherland** stressed the need for more widespread adoption of flying squads in spite of difficulties at Regional Board level.

**Mr. Bruce Blair** proposed a vote of thanks to Mr. Stallworthy and said how much he had been impressed by the clarity and simplicity of the address. He felt it was of the greatest value to the branch that Mr. Stallworthy should visit them and he felt sure that all were extremely grateful for his trouble in coming from Oxford.

